# Proteomic Studies of a Single CNS Synapse Type: The Parallel Fiber/Purkinje Cell Synapse

**DOI:** 10.1371/journal.pbio.1000083

**Published:** 2009-04-14

**Authors:** Fekrije Selimi, Ileana M Cristea, Elizabeth Heller, Brian T Chait, Nathaniel Heintz

**Affiliations:** 1 Laboratory of Molecular Biology, Howard Hughes Medical Institute, The Rockefeller University, New York, New York, United States of America; 2 CNRS, UMR7102, Paris, France; 3 UPMC, UMR7102, Paris, France; 4 Laboratory for Mass Spectrometry and Gaseous Ion Chemistry, The Rockefeller University, New York, New York, United States of America; 5 Department of Molecular Biology, Princeton University, Princeton, New Jersey, United States of America; Baylor College of Medicine, United States of America

## Abstract

Precise neuronal networks underlie normal brain function and require distinct classes of synaptic connections. Although it has been shown that certain individual proteins can localize to different classes of synapses, the biochemical composition of specific synapse types is not known. Here, we have used a combination of genetically engineered mice, affinity purification, and mass spectrometry to profile proteins at parallel fiber/Purkinje cell synapses. We identify approximately 60 candidate postsynaptic proteins that can be classified into 11 functional categories. Proteins involved in phospholipid metabolism and signaling, such as the protein kinase MRCKγ, are major unrecognized components of this synapse type. We demonstrate that MRCKγ can modulate maturation of dendritic spines in cultured cortical neurons, and that it is localized specifically to parallel fiber/Purkinje cell synapses in vivo. Our data identify a novel synapse-specific signaling pathway, and provide an approach for detailed investigations of the biochemical complexity of central nervous system synapse types.

## Author Summary

The brain is composed of many different types of neurons that form very specific connections: synapses are formed with specific cellular partners and on precise subcellular domains. It has been proposed that different combinations of molecules encode the specificity of neuronal connections, implying the existence of a “molecular synaptic code.” To test this hypothesis, we describe a new experimental strategy that allows systematic identification of the protein composition for individual synapse types. We start with mice that are genetically engineered to facilitate the purification of one type of synapse from a given neuronal population in the central nervous system, the parallel fiber/Purkinje cell synapse. The purification is performed using a combination of biochemical fractionation and affinity purification. Subsequent mass spectrometry allows us to identify approximately 60 different proteins present in the resulting sample. We have further analyzed some of the 60 proteins and show that MRCKγ, a newly identified kinase, is localized in the dendritic spines where the parallel fiber/Purkinje cell synapses are formed and that it can modulate the morphogenesis of dendritic spines. The use of this experimental strategy opens up the ability to provide insights into the underlying “molecular code” for the diverse types of synapses in the brain.

## Abstract 3

In a first step toward characterizing a putative molecular "synaptic code," a strategy--using genetically modified mice and cell fractionation to purify one class of synapses followed by mass spectrometric protein identification--reveals new proteins at the parallel fiber/Purkinje cell synapse.

## Introduction

Each of the thousands of cell types present in the nervous system receives multiple classes of inputs that are spatially segregated and functionally distinct. The chemoaffinity hypothesis stated that “the establishment and maintenance of synaptic associations were conceived to be regulated by highly specific cytochemical affinities… .” [1]. Support for this idea has come from studies of specific synaptic proteins [2,3]. For example, different sets of neurotransmitter receptors are found at different synapse types [3], even at excitatory synapses made on the same neuron [4]. Precise subcellular targeting of synapses is also dependent on the recognition of specific molecules such as adhesion proteins [5]. In addition to these direct-recognition mechanisms, guidepost cells seem to target synapse formation to precise locations: their role has been demonstrated in both invertebrates [6] and vertebrates [7]. Synaptic physiology is also regulated by mechanisms that are synapse type-dependent, since similar stimulation patterns can have opposite effects on plasticity of different synapses [8]. Therefore, the formation and function of each type of synapse is controlled by a complex activation of signaling pathways through specific proteins.

Since the visualization of synapses by electron microscopy, attempts have been made at biochemically purifying them and at identifying their chemical composition, especially for the postsynaptic densities characteristic of excitatory synapses [9,10]. The use of mass spectrometry (MS) to identify proteins in complex mixtures has greatly improved our ability to unravel the protein composition of organelles. Using this technique, over 1,000 different postsynaptic proteins have been identified in “bulk” postsynaptic density preparations or in affinity-purified receptor complexes [11–16]. These proteins have a wide range of functions: receptors to neurotransmitters, scaffold proteins, kinases, enzymes, etc. Recently, combining comparative genomics and proteomics, Emes and collaborators [17] have shown that increased behavioral complexity correlates with a phylogenetic expansion of synaptic proteins that are involved in upstream signaling pathways, such as receptors and adhesion molecules. Microarray analysis also showed a very variable regional expression pattern for these upstream synaptic proteins [17], in accordance with previously obtained results for neurotransmitter receptors [3]. The complexity of the synaptic proteome illustrated by these data highlights the need for studies aimed at systematically identifying the protein composition of individual synapse types, and understanding their mechanistic diversity.

To address this issue, we have developed synaptic protein profiling as an approach to isolate and biochemically characterize specific types of central nervous system (CNS) synapses. We chose to analyze first the parallel fiber to Purkinje cell (PF/PC) synapse in the cerebellum, because of its unique physiological properties and its involvement in neurological disease [18,19]. We engineered mice to tag and purify specifically PF/PC synapses and, using MS, we have identified 65 proteins located at the PF/PC synapse. This dataset provides clues to PF/PC synapse-specific signaling pathways, as illustrated by our functional analysis of one of these proteins, MRCKγ. Our results provide an important example of the biochemical complexity of an individual synapse type, and reveal a new mechanism for the regulation of synaptic function.

## Results

### Purifying the Parallel Fiber/Purkinje Cell Synapse

**Figure 1:**
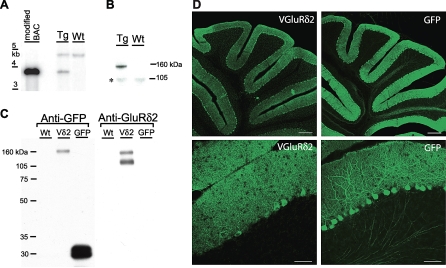
Tagging the Parallel Fiber/Purkinje Cell Synapse in Transgenic Mice (A) Southern blot was used to identify transgenic mice having integrated the Pcp2 BAC (a Purkinje cell–specific driver) containing the Venus-tagged GluRδ2 receptor, VGluRδ2. Tg, transgenic; Wt, wild type. (B) VGluRδ2 expression was detected using an anti-GFP antibody on immunoblots from total protein extracts of transgenic (Tg) versus wild-type (Wt) cerebella. An asterisk (*) indicates a nonspecific band. (C) Both VGluRδ2 and GFP were affinity purified using an anti-GFP antibody from 1% Triton X-100 cerebellar extracts from wild-type (Wt), Pcp2/VGluRδ2 (Vδ2), and Pcp2/eGFP control (GFP) mice, as shown by probing the immunoblots with an anti-GFP antibody (left). VGluRδ2 specifically copurified the endogenous GluRδ2, as shown by probing the same blot with an anti-GluRδ2 antibody (right). (D) Immunofluorescence on cerebellar sections using an anti-GFP antibody shows the specific localization of VGluRδ2 in the molecular layer and somata of Purkinje cells of Pcp2/VGluRδ2 mice. Soluble GFP is detected in the molecular layer, dendrites, somata, and axons of Purkinje cells in sections from Pcp2/eGFP mice. Scale bars in the upper panels indicate 200 μm; lower panels, 50 μm.

**Figure 2:**
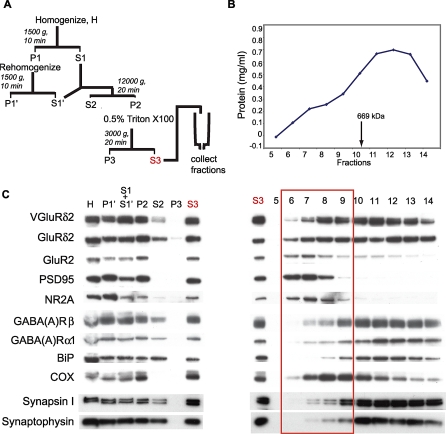
VGluRδ2 Is Detected in Excitatory Synaptic Fractions Using a New Purification Method (A) We prepared a crude synaptosome P2 fraction that was solubilized in 0.5% Triton X-100 final concentration. The extract was then separated on a Sephacryl S1000 gel filtration column. Calibration of the column indicated that protein complexes smaller than 669 kDa (arrow in [B]) were resolved after fraction 10. (B) Protein dosage was performed on every fraction collected. (C) Each fraction (0.1% in volume) was run on western blots and assayed for the presence of excitatory postsynaptic markers (GluRδ2, GLUR2, PSD95, and NR2A), presynaptic markers (synapsin I and synaptotagmin), inhibitory synapse markers (GABA(A)Rβ and GABA(A)Rα1), the endoplasmic reticulum marker BiP, and the mitochondrial marker COX. VGluRδ2 was detected using an anti-GFP antibody. The red rectangle outlines the “excitatory synaptic” fractions enriched for synaptic markers and pooled for subsequent affinity-purification of PF/PC PSDs.

To enable purification of PF/PC synapses, we developed a transgenic line that expresses an affinity tag only at the PF/PC synapse. We generated a fusion between the glutamate receptor delta2, GluRδ2 (National Center for Biotechnology Information [NCBI]# EDK98768), which is specifically localized at the PF/PC postsynaptic density [20], and Venus, a variant of the green fluorescent protein (GFP). The resulting fusion protein, VGluRδ2, is properly processed and transported to the cell surface (Figure S1). To express the fusion specifically in cerebellar Purkinje cells, the VGluRδ2 cDNA was then incorporated into a Pcp2 bacterial artificial chromosome (BAC) by homologous recombination, and the resulting Pcp2/VGluRδ2 BAC construct was used to generate transgenic mice (Figures 1A and S1). Expression of the fusion polypeptide was detected in the cerebellar extracts of Pcp2/VGluRδ2 transgenic mice (Figure 1B), and coimmunoprecipitation experiments demonstrated proper assembly of the VGluRδ2 fusion with the endogenous GluRδ2 receptor subunits (Figure 1C). As shown in Figure 1D, the localization of VGluRδ2 in the molecular layer and somata of PCs agrees with the synaptic localization of the GluRδ2 receptor. In contrast, the enhanced GFP (eGFP) control protein expressed using the same BAC vector (Pcp2/eGFP; http://www.gensat.org) is detected throughout the cell, including marked labeling of both Purkinje cell dendrites and axons (Figure 1D). Prior to the affinity-purification step, we sought to produce cerebellar extracts enriched for synaptic structures relative to trafficking complexes, and to maximize the recovery of VGluRδ2-tagged postsynaptic densities (PSDs). This was performed by fractionation of a solubilized crude synaptosome fraction (S3) on a gel-filtration column (Figure 2A). As shown in Figure 2B and 2C, this resulted in an enrichment of postsynaptic and mitochondrial proteins, and a relative depletion of endoplasmic reticulum (ER) components and presynaptic proteins in the high molecular weight fractions. These excitatory synaptic fractions contain essentially all of the PSD95 scaffolding protein. They also contain VGluRδ2, which was distributed amongst the different fractions in the same manner as wild-type GluRδ2 (Figure 2C). This was also observed using a standard synaptosome purification (Figure S2) and shows that the fusion receptor VGluRδ2 is targeted to the synapse similarly to the wild-type GluRδ2.

**Figure 3:**
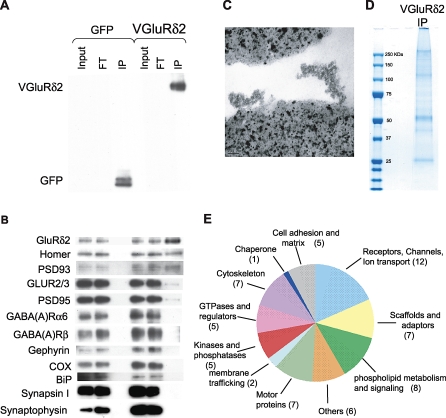
Affinity Purification and Protein Profiling of the Parallel Fiber/Purkinje Cell PSDs (A) Synaptic fractions from Pcp2/VGluRδ2 animals were affinity purified using magnetic beads coated with anti-GFP antibody (VGluRδ2). In parallel, control purifications were performed on preparations from Pcp2/eGFP transgenic mice (GFP); 0.025% of the inputs and flow-throughs (FT), and 25% of the affinity-purified samples (IP) were assayed by western blot using an anti-GFP antibody and showed immunoprecipitation of both VGluRδ2 and GFP, respectively. (B) The same blot was probed for different postsynaptic markers, presynaptic markers (synapsin I and synaptotagmin), the mitochondrial protein COX, and the ER marker BiP, showing specific copurification of postsynaptic markers localized to the PF/PC synapse. (C) Electron microscopy shows the presence of electron-dense structures reminiscent of PSDs on the surface of the magnetic beads used for affinity purification of Pcp2/VGluRδ2 extracts. (D) Proteins from the affinity-purified VGluRδ2 PSDs were separated by SDS-PAGE electrophoresis and stained with Coomassie Blue before MS analysis. (E) MS identified 65 different proteins in the complexes purified from Pcp2/VGluRδ2 mice. These proteins can be classified into 11 functional categories. The number of proteins from each category is indicated in parentheses. Nonshaded areas represent proteins found with high confidence.

To separate PF/PC PSDs from other cerebellar synapses, we performed affinity purification from the pooled excitatory synaptic fractions (red rectangle, Figure 2C) using an anti-eGFP antibody. Electron microscopy of the affinity purified material showed electron-dense structures that were reminiscent of PSDs [21] on the surface of the beads used for purification of VGluRδ2 extracts (Figures 3C and S3). These structures were absent from beads used to immunopurify extracts from Pcp2/eGFP control cerebella.

Using western blots, we could show that more than 50% of the target protein was immunopurified from the input extract for either the control eGFP or the VGluRδ2 extracts (Figure 3A and unpublished data). Western blotting also demonstrated copurification of several PF/PC synaptic components with VGluRδ2, including the GluRδ2 and GluR2/3 receptors, and the scaffolding proteins PSD93 and Homer (Figure 3B). Markers of inhibitory synapses (GABA(A) receptor α6, GABA(A) receptor β, and gephyrin), presynaptic structures (synapsin I and synaptophysin), or of mitochondria (Cox) did not copurify, demonstrating the specificity of this approach (Figure 3B). As expected, none of these markers copurified with soluble eGFP in extracts prepared from Pcp2/eGFP control mice (Figure 3B). Taken together, these results demonstrate that the combination of cell-specific genetic targeting, molecular tagging of specific CNS synapses, biochemical fractionation, and affinity purification can be used to enrich for a specific type of PSD from crude brain extracts.

### Sixty-Five Proteins Identified in Purified Parallel Fiber/Purkinje Cell Postsynaptic Densities

To systematically identify components of the PF/PC PSDs, we analyzed the protein content of pooled PF/PC PSD preparations using single- and two-stage MS [22]. A first sample, prepared by pooling three experiments using ten Pcp2/VGluRδ2 cerebella each, enabled us to identify 12 components present at the PF/PC synapse but not present in the control sample prepared in parallel from Pcp2/eGFP cerebella (Table S1). To increase the number of PF/PC PSD components identified, we performed a second analysis on a sample prepared with a total of 50 cerebella (Figure 3D). A total of 65 proteins were identified: 37 proteins were detected with high confidence (Figures S4 and S5; Tables S1 and S3), and 28 were observed at lower levels and identified with less confidence (Figure S6; Tables S2 and S4). This analysis confirmed the presence of the PF/PC synapse proteins GluRδ2 [23], Homer-3 [24], PSD93 [23], delphilin [23], Shank1, and Shank2 [25], and the absence of proteins located at other excitatory (NMDA receptor subunits, GABA(A) receptor α6) or inhibitory synapses (GABA(A) receptor α6, GABA(A) receptor β, and gephyrin) in the cerebellum. Forty of the identified proteins in our affinity-purified PSDs have been previously detected in preparations of synaptic proteins ([16] and Tables S1 and S2).

The 65 proteins we have identified can be grouped into 11 different functional categories (Figure 3E; Tables S1 and S2). These categories have been previously annotated in studies of the postsynaptic density [13], with the exception of a class of proteins that we have called “phospholipid metabolism and signaling.” In the “scaffolds and adaptors” category, several members of the Shank family (1 and 2) and the PSD family (PSD93 and PSD95) were detected at the PF/PC synapse, illustrating redundancy for scaffold proteins, certainly due to their importance for synaptic function. Other functional categories include proteins important for synapse formation and physiology, such as regulators of small GTPases and protein kinases. Interestingly, eight of the proteins identified in our study can regulate or be regulated by phospholipid metabolism (Iptr1, synaptojanin 1 and 2, phospholipase B, ABCA12, and MRCKγ), or contain phospholipid-binding domains (Plekha7, annexin A6, and MRCKγ), and were thus grouped into a previously unrecognized category “phospholipid metabolism and signaling.” This suggests that phospholipid regulation is a major feature of the PF/PC synapse. Another important category present at synapses groups receptors and ion channels: several glutamate receptor subunits and several G protein–coupled receptors (GABA-B and BAI receptors) were detected in our analysis of the PF/PC PSD. Interestingly, the extracellular domain of BAI receptors contains thrombospondin repeats, which can mediate cell adhesion [26]. Several other proteins identified at the PF/PC synapse in our study are involved in cell adhesion and interaction with the extracellular matrix: receptor protein tyrosine phosphatases [27], delta-catenin-2 [28], Neph1 [29] and laminins [30]. These diverse potential recognition proteins could form together a “code” defining the PF/PC synapse.

**Figure 4:**
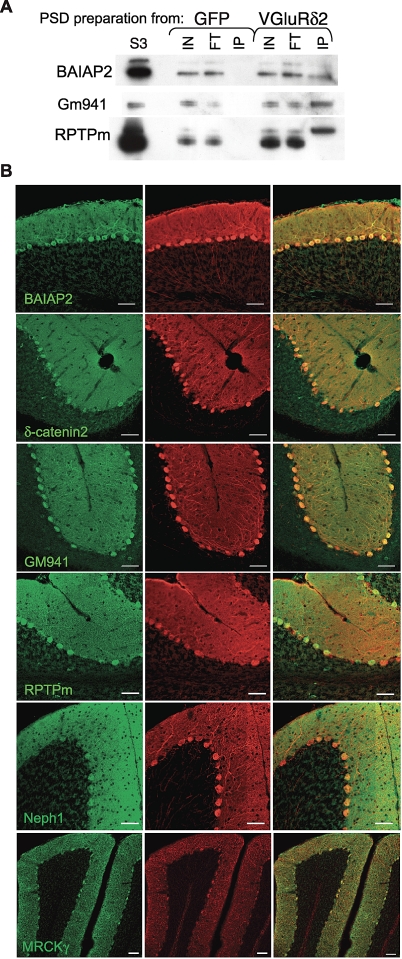
Localization of Several Candidate Synaptic Proteins at the Parallel Fiber/Purkinje Cell Synapses (A) Presence of selected candidates in PF/PC PSDs purified from Pcp2/VGluRδ2 cerebella; 0.025% of the inputs (IN) and flow-throughs (FT), and 25% of the affinity-purified samples (IP) obtained from Pcp2/eGFP control (GFP) and Pcp2/ VGluRδ2 (VGluRδ2) cerebella were assayed by western blot. (B) Localization by immunofluorescence of candidate synaptic proteins. Labeling was performed using antibodies recognizing several candidate proteins identified by MS (green) in conjunction with an anti-calbindin antibody specifically labeling Purkinje cells (red). Scale bars indicate 50 μm.

To provide additional evidence for the synaptic localization of the novel components that we have identified, we performed immunofluorescence studies on cerebellar sections from wild-type mice. Localization in the molecular layer of the cerebellum, which contains the PF/PC synapses, was evident for MRCKγ, Gm941, BAIAP2, RPTPm, Neph1, and delta2-catenin (Figure 4). Delta2-catenin and Gm941 could also be detected in some cerebellar interneurons. We also examined the expression of candidates reported in in situ hybridization databases (http://www.stjudebgem.org; http://www.brain-map.org; and http://www.genepaint.org). Interpretable data were available for 42 candidates, and all but two were expressed in Purkinje cells, with a majority showing little detectable expression in the granule cell layer (Tables S1 and S2). These expression data provide additional confirmation that the majority of the proteins identified in our study are bona fide components of the PF/PC synapse.

### MRCKγ: An Example of a New Signaling Pathway at the Parallel Fiber/Purkinje Cell Synapse

**Figure 5:**
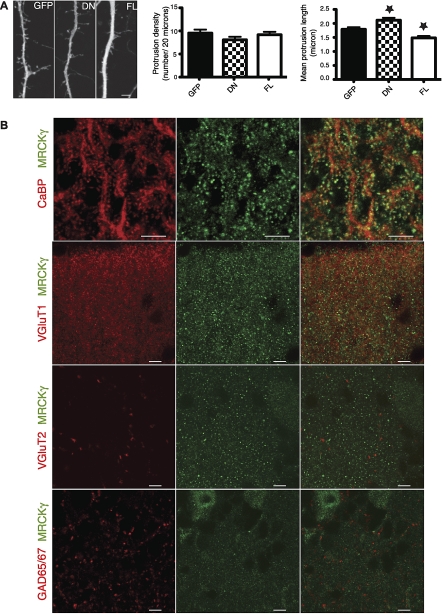
MRCKγ Modulates Dendritic Spine Morphogenesis and Is Localized to Parallel Fiber/Purkinje Cell Spines (A) Primary cortical cultures were imaged at DIV14 after transfection with either GFP alone or in conjugation with MRCKγ lacking the kinase domain (DN) or the full-length MRCKγ kinase (FL) (left panel, scale bar indicates 10 μm). Mean protrusion density was not significantly different between the three conditions (middle), but significant changes in mean protrusion length were observed (right panel, asterisk [*] indicates *p* < 0.05 compared to GFP). (B) Immunofluorescence labeling shows the localization of MRCKγ (green) in Purkinje cell spines (calbindin, red) and its colocalization with VgluT1, a marker of parallel fiber synapses, but not with VgluT2 or GAD65/67, markers of climbing fiber or of inhibitory synapses respectively. Scale bars indicate 5 μm.

Within our “phospholipid metabolism and signaling” category, we identified the kinase MRCK gamma (MRCKγ, NCBI# Q80UW5), which has not previously been localized to synapses. Since MRCK family members have been shown to regulate cytoskeleton reorganization and cell morphology [31,32], we sought to test the role of MRCKγ in dendritic spine morphogenesis in primary cortical cultures. Comparative analysis of dendritic protrusions was carried out for cultures transfected either with GFP alone, or in combination with full-length MRCKγ (MRCKγFL) or a MRCKγ construct lacking the kinase domain (MRCKγDN) (Figure 5A). Protrusion density is not significantly affected by overexpression of either form of the kinase (GFP: 9.5 ± 0.7 protrusions per 20 μm; MRCKgDN: 8.0 ± 0.6; MRCKgFL: 9.1 ± 0.6; one-way ANOVA, *p* = 0.29). However, the mean length of dendritic protrusions in neurons overexpressing MRCKγFL decreased when compared to control neurons, whereas the length of protrusions in MRCKγDN-transfected neurons increased (GFP: 1.79 ± 0.07 μm; MRCKgDN: 2.11 ± 0.08; MRCKgFL: 1.48 ± 0.06; *p* < 0.05 for all comparisons, Dunn multiple comparison test). The effect of MRCKγDN implies that it can interfere with endogenously expressed MRCK kinases. Indeed, after data mining of previously published results, we found that MRCKβ has been identified in “bulk” PSD preparations from mouse brain, and thus could be present in cortical neurons [16]. Since mean spine length decreases with maturation [33], our data demonstrate that MRCK family members, through their kinase function, increase maturation of dendritic spines in primary CNS neurons.

Given the presence of MRCKγ in our PSD preparations, and its ability to modulate dendritic spine morphogenesis, we were next interested in its subcellular localization in Purkinje cells (Figure 5B). High-resolution confocal immunofluorescence using an antibody against MRCKγ clearly demonstrated its presence in Purkinje cell dendritic spines, which have been shown to contain GluRδ2 [4]. Moreover, colabeling with markers of specific synapses on Purkinje cells showed that MRCKγ is extensively colocalized with VGluT1, a marker for PF/PC synapses. MRCKγ is not present in structures labeled by VGluT2 or GAD65/67, which are present at climbing fiber and inhibitory Purkinje cell synapses, respectively. Taken together, our data support a specific role for MRCKγ in the maturation and plasticity of PF/PC synapses, and confirm the importance of synapse-specific protein profiling for the discovery of signaling pathways that modulate the development and function of specific CNS synapse types.

## Discussion

We have demonstrated that the biochemical components of a specific synapse type from a particular neuronal population can be identified using a combination of genetically engineered mice, affinity purification, and MS. Using our approach, we have prepared a fraction enriched in PF/PC PSDs and identified 65 proteins classified in 11 different functional categories. This dataset provides information on signaling pathways specifically tethered to this synapse, as exemplified by our functional analysis of MRCKγ. It also provides information on the variety of proteins that can be part of the code defining the PF/PC synapse.

Approximately 700 different proteins have been identified in PSD preparations from whole brain [16]. However, it has been estimated that, given the mass of a single PSD, the copy number of scaffold proteins in a PSD, and an average size of 100 kDa for each synaptic protein, only about 100 different proteins can be expected to be found at one particular type of PSD [34]. The number of proteins we find in our study is consistent with that estimate. Although our analysis may not have revealed all PF/PC postsynaptic proteins, the successful identification of AMPA receptor subunits in our preparations suggests that any proteins not detected in our sample must be present at low stochiometries in the PSD.

Synaptic protein profiling can reveal novel sets of proteins that allow formulation of specific hypotheses regarding synaptic function. For example, we discovered MRCKγ at PF/PC synapses: this kinase is part of a family that has never been described at synapses. This result was striking since MRCK proteins can respond to small GTPases signaling and have been shown to modulate actin cytoskeleton and cell morphology in nonneuronal systems [31]. These characteristics immediately suggest a role for these kinases in spine morphogenesis, which we have now shown for MRCKγ using transfection of cultured cortical neurons. Taken together, these data also have implications for the study of neurodevelopmental diseases. Deficiencies in spine length and spine morphology in Purkinje cells have been found in models of mental retardation and Angelman syndrome [35,36]. Given the link between another small GTPase-dependent kinase, PAK3, and mental retardation [37], our results suggest that MRCKγ could participate in the signaling pathways involved in mental retardation and autism spectrum disorders.

Another interesting finding of our study is the presence of a high proportion of proteins involved in phospholipid metabolism and signaling at the PF/PC PSD. A major regulator of the physiology of the PF/PC synapse is the metabotropic glutamate receptor 1 (mGluR1) which induces phosphatidylinositol-4,5-P2 (PIP2) hydrolysis through activation of phospholipase C [8,18]. Our results show the presence of MRCKγ and Itpr1 in affinity-purified PF/PC PSDs: these proteins can respond to, respectively, DAG and IP3, which are the metabolites of PIP2 hydrolysis. This further supports the importance of mGluR1 signaling at the PF/PC synapse and extends the number of regulatory pathways potentially activated by mGluR1. Also included in the “phospholipid signaling and metabolism” category in our data are synaptojanin-1 and −2, two PIP2-metabolizing enzymes. These enzymes are best known for their regulation of vesicle recycling at synapses, but have also been found by other biochemical studies at PSDs [16]. Phospholipid metabolism is known to be critical for the function of the presynaptic side of the synapse, especially vesicle recycling [38]. It also plays a role in defining the boundaries of the apical pole and the localization of tight junctions in epithelial cells [39]. Our results suggest that phospholipid signaling also participates in regulating the structure and stability of PSDs. Given the fact that lithium is used as a treatment for schizophrenia and bipolar disorders, and that it might act by modulating phospholipids' metabolism [40], our results may be particularly relevant for studies of a variety of human neurological disorders. Indeed, it has been suggested that synaptojanin-1 is involved in the cognitive defects observed in Down syndrome [41], and that PIP2 metabolism may be linked to synaptic dysfunctions in Alzheimer disease [42].

The results presented here provide clues to the nature of the “synaptic code” and the types of molecules that may be critical in definition of specific synapse types. As expected from previous studies [2], we find proteins with classical adhesion domains such as Neph1 and the receptor tyrosine phosphatase RPTPmu. SYG1, the Caenorhabditis elegans homolog of Neph1, has been shown to define synapse location in vivo [6], and may play a similar role for the PF/PC synapse. Receptor tyrosine phosphatases play important roles in axon guidance, and have also been shown to control synapse formation [43]. We also find proteins at the PF/PC synapse with as yet unknown functions in synaptogenesis, such as the BAI receptors or GABA-B receptors. In this regard, it is interesting to note that the GABA-B receptor 1 contains a CCP module in its extracellular domain. This module is also found in proteins of the complement cascade, which have recently been shown to be involved in synapse development [44]. These proteins, and the majority of the remaining proteins identified in this study, are specifically expressed in Purkinje cells within the cerebellum (see Results). Since cerebellar granule cells also receive excitatory inputs from mossy fibers, we can conclude that, even within the cerebellum, the synaptic codes for specific synapse types must be quite distinct. This supports the results of expression analysis of proteins identified in bulk synapse preparations showing that receptors and other upstream signaling molecules have a highly variable expression pattern in the vertebrate brain [17]. Taken together, these data indicate that very different sets of molecules must define different excitatory synapse types.

Although our approach employed the expression of a fusion of GluRδ2 with EGFP in a specific cell type, this basic approach can readily be adapted to characterize a wide variety of synapse types, given the wide range of affinity tags that are now available and the hundreds of BAC vectors that can be used to target expression to specific neurons (http://www.gensat.org). We anticipate that these additional studies of the biochemical diversity of synapses will be critical for understanding the development and function of specific CNS circuits and their dysfunction in disease [45,46].

## Materials and Methods

### Animals.

All experiments using animals were performed according to protocols approved by the Institutional Animal Care and Use Committee at the Rockefeller University. Both the Pcp2/eGFP and the Pcp2/VGluRδ2 transgenics were bred on the FVB background, and littermates were used as wild-type controls.

### Preparation of PSDs and affinity purification.

Ten cerebella from adult mice were used for the preparation of a crude synaptosome fraction P2 as presented in Figure 2A (based on previously published protocols [47]). The solution used as a homogenization and resuspension buffer contained 0.32 M sucrose, 5 mM HEPES, 0.1 mM EDTA (pH 7.3), and a protease inhibitor cocktail (Sigma). P2 was then solubilized 30 min at 4 °C using a final concentration of 0.5% Triton X-100. The cleared solubilized fraction was separated by gravity flow on a gel-filtration column (Sephacryl S1000 Superfine; GE Healthcare) prepared using a solution containing 2 mM CaCl_2_, 132 mM NaCl, 3 mM KCl, 2 mM MgSO_4,_ 1.2 mM NaH_2_PO_4_, 10 mM HEPES, and 0.5% Triton X-100 (pH 7.4). 2-ml fractions were collected, and aliquots were used for protein dosage using the BCA Protein assay kit (Pierce Biotechnology). Calibration of the gel-filtration column was performed using the gel-filtration HMW calibration kit (GE Healthcare).

Pooled fractions from the column were used for affinity purification of tagged PSDs. Dynabeads M-270 epoxy beads (Dynal) were conjugated using 15 μg of affinity-purified goat anti-GFP antibody per milligram of beads [22]; 6 mg of beads were used for affinity purification of pooled synaptic fractions from ten cerebella during 1 h at 4 °C. Beads were then washed in 2 mM CaCl_2_, 300 mM NaCl, 3 mM KCl, 2 mM MgSO_4_, 1.2 mM NaH_2_PO_4_, 10 mM HEPES, and 0.5% Triton X-100. Purified complexes were finally eluted in 0.5 N NH_4_OH, 0.5 mM EDTA for 20 min, dried, and then resuspended in the desired volume of protein electrophoresis sample buffer. Biochemical preparations and affinity purifications were performed in parallel for each genotype, starting with ten cerebella each. For MS analysis, samples from several successive experiments were pooled.

### Mass spectrometry analysis.

Following immunopurification, the isolated proteins were resolved by 1-D SDS-PAGE and stained with Coomassie Blue (GelCode Blue; Pierce). As proteins stain with varied efficiency, for each sample (from the 30- and 50-mice preparations), the complete gel was subjected to mass spectrometric analysis. Each entire gel lane was cut into 66 × 1 mm sections. The 1-mm sections were combined in approximately 30 samples, and proteins were digested with 12.5 ng/μl sequencing-grade modified trypsin (Promega). The resulting peptides were extracted on reverse-phase resin (Poros 20 R2; PerSeptive Biosystems) and eluted with 50% (v/v) methanol, 20% (v/v) acetonitrile, and 0.1% (v/v) trifluoroacetic acid containing 2,5-dihydroxybenzoic acid (2,5-DHB; 1:3 v/v saturated matrix solution in elution solution). Samples were subjected to matrix-assisted laser desorption/ionization (MALDI) quadrupole/time-of-flight (QqTOF) MS and MALDI ion trap (MALDI-IT) tandem MS (MS/MS) analyses using an in-house–built MALDI interface coupled to a Qq-TOF instrument (QqTOF Centaur; Sciex) and an ion trap (LCQDECAXP^PLUS^; Finnigan) as described [22,48,49]. XProteo computer algorithm (http://www.xproteo.com) was used to search the peptide fingerprint data and collision-induced dissociation (CID) MS/MS data in the NCBI database (see Text S1). Due to the limited amount of samples, all MALDI-IT CID MS/MS spectra were carefully acquired and interpreted manually. A MS/MS hypothesis-driven approach on isolates from control Pcp2/eGFP transgenic mice was used to probe for the specificity of the proteins copurified with VGluRδ2 (Text S1).

### Dendritic spine morphometric analysis.

The MRCKγ cDNA was amplified from cerebellar cDNA. The MRCKγDN construct was obtained by deleting the sequence encoding for amino acids 1–426, and by replacing it with ATG. MRCKγ and eGFP cDNAs were subcloned in the bidirectional Tet-responsive vector pBI (Clontech).

Primary neuronal cultures were prepared from E15 mouse embryos (Swiss strain). Cortices were dissected and triturated using a fire-polished Pasteur pipette and 0.05% trypsin. Neurons were plated on poly-d-lysine and laminin-coated coverslips at a density of 1.5 × 10^5^ cells/cm^2^ and cultured in neurobasal medium supplemented with 2% B27 supplement, 0.5 mM glutamine, and antibiotics. Transfections were performed at DIV7 with a 1:1 ratio of a tTA-expressing plasmid and the bidirectional vector containing GFP (with or without the kinases) using Lipofectamine 2000 according to the manufacturer's instructions (Invitrogen).

Dendrites of transfected neurons were imaged using a confocal microscope and a 63× objective with a 5× zoom. Quantifications of protrusion density and length were performed using the NeuronJ plugin and the ImageJ software on several dendrites per neuron (at least five different cells per transfection condition, four independent experiments). A total of 1,029, 861, and 950 protrusions were counted and measured for the GFP, DN, and FL transfections, respectively. Statistical analysis was performed using the GraphPad Prism software.

## Supporting Information

Figure S1 Construction of a Fusion between Venus and GluRδ2 (VGluRδ2) (A) Venus was fused on the N-terminal extracellular part of GluRδ2 (top left panel). A GluRδ2-positive band was detected in protein extracts from VGluRδ2-transfected HEK293 cells, but not in extracts from Venus-transfected cells (bottom left panel). The band was at the expected size (about 140 kDa), higher than the endogenous GluRδ2 detected in cerebellar extracts. Immunofluorescence using an anti-GFP antibody detected the extracellular Venus in VGluRδ2-transfected cells in nonpermeabilizing conditions (red, right panels), showing the proper topography of the tagged receptor.
(B) The correct modification of the Pcp2 BAC with the VGluRδ2 construct was checked by Southern blot (left panel, probe shown in [C], BAC DNA digested with EcoRI) and pulse-field gel electrophoresis (right panel, BAC DNA digested with SpeI), before injection in mouse oocytes.
(C) Schematic diagram of the BAC containing the Pcp2 gene, known to be expressed specifically in Purkinje cells. The VGluRδ2 cDNA was placed at the level of the Pcp2 ATG. The arrow indicates the promoter region.
(463 KB AI).

Figure S2 VGluRδ2 Is Fractionated Similarly to the Wild-Type GluRδ2 Receptor Using a Classical Synaptosome Preparation Fractions obtained using the protocol of Dunkley et al. [47] for synaptosome preparation were probed for excitatory synapse markers (GluRδ2, PSD95), the inhibitory synapse marker GABA(A)Rα1, the endoplasmic reticulum marker BiP, and the mitochondrial marker COX. VGluRδ2 was detected using an anti-GFP antibody.
(498 KB AI).

Figure S3 Immunoelectron Micrograph of Affinity-Purified PSDs from VGluRδ2 Cerebella Labeled with an Anti-PSD95 Antibody (9.05 MB AI).

Figure S4 Example of the Mass Spectrometric Strategy Utilized for Protein Identification and Confirmation, Illustrated for PSD93 and PSD95 (A) Following in-gel digestion with trypsin, the mixture of peptides was analyzed by MALDI QqToF MS. The m/z values of the [M+H]+ peptides were searched in the NCBI database using the XProteo software, and PSD93 and PSD95 were the first two hits with high scores. The lists of putative peptides generated by the XProteo software are shown, and their presence in the MALDI QqToF MS is indicated. T, trypsin peptides.
(B) The identity of the proteins was confirmed using MALDI-IT CID MS/MS analyses, and their specificity of isolation was investigated using a hypothesis-driven tandem MS approach on preparations from Pcp2/eGFP transgenic mice (GFP). Examples of results from MS/MS analyses on peptides of both high- and low-signal-to-noise ratios are shown.
(1.55 MB TIF)

Figure S5 The Analysis of Internexin and Camk2b in Immunoaffinity Purifications of VGluRδ2 Exemplifies the Identification and Confirmation of Proteins That Were Not Assigned a Score after the Database Search Using the XProteo Software (A) Internexin and Camk2b peptides were both observed following MALDI QqToF MS analysis; however, only internexin received an XProteo database search score (d′ = 6).
(B) The presence of both internexin and Camk2b was confirmed using MALDI-IT CID MS/MS analyses.
(868 KB TIF)

Figure S6 Examples of Spectra Obtained for Low-Confidence Candidates Representative MALDI-IT CID MS/MS spectra are shown for Atp1a1 and Ncoa7. MALDI QqToF MS data and list of putative peptides are illustrated for Ptprm. The peaks attributed to Ptprm are shown with orange arrowheads. This portion of the gel contained multiple proteins. Light blue and dark blue dots indicate selected peaks attributed to Fodrin alpha chain and traces of GluRδ2, respectively. GluRδ2 was primarily identified in another gel band. Grey dot indicates a heavy labeled GluRδ2 peptide, spiked in all samples containing GluRδ2.
(823 KB TIF)

Table S1 List of Proteins Identified with Higher Confidence in the Immunoisolates of Venus-Tagged GluRδ2 Functional category, expression in Purkinje cells (PCs), and previous identification in PSD preparations (@PSD) are given.
(a) Reference numbers in this column refer to the list in Text S1.
(b) From reference [16]. Y indicates that the protein is detected; N, not detected; and I, the isoform is detected.
(27 KB XLS)

Table S2 List of Proteins Identified in the Immunoisolates of Venus-Tagged GluRδ2 with Lower Levels of Confidence as Judged by Mass Spectrometry Functional category, expression in Purkinje cells (PCs), and previous identification in PSD preparations (@PSD) are given.
(a) Reference numbers in this column refer to the list in Text S1.
(b) From reference [16].
Y indicates that the protein is detected; N, not detected; and I, the isoform is detected.
(26 KB XLS)

Table S3 List of Proteins Identified in the Immunoisolates of Venus-Tagged GluRδ2 Results are shown of two replicate experiments from either 30 or 50 mice. The detection and confirmation of the proteins through MS and MS/MS analyses are indicated for both experiments. The sequence coverage, number of peptides, and scores obtained from the analysis of the MALDI QqToF MS spectra are given for the 50-mice experiment. The number and sequences of peptides confirmed by MALDI-IT CID MS/MS analyses are shown for each protein. The presence of these proteins in the control experiment, as judged by hypothesis-driven MS/MS analyses, is indicated. When the presence or absence of the protein could not be judged conclusively, due to either depletion of the sample or inconclusive fragmentation, the entry is marked as not available (n/a). n/o (not observed) in the score column refers to the proteins (MS) or peptides (MS/MS) that were not assigned a d′ value from the database search using the XProteo software.
(57 KB XLS)

Table S4 List of Proteins Identified in the Immunoisolates of Venus-Tagged GluRδ2 with Lower Levels of Confidence as Judged by Mass Spectrometry Results are shown from two replicate experiments from either 30 or 50 mice. The detection and confirmation of the proteins through MS and MS/MS analyses is indicated for both experiments. The sequence coverage, number of peptides, and scores obtained from the analysis of the MALDI QqToF MS spectra are given for the 50-mice experiment; a caret (∧) indicates a score obtained only after searching manually in the MS spectrum for peptides that could correspond to the protein of interest. The number and sequences of peptides confirmed by MALDI-IT CID MS/MS analyses are shown for each protein. Several proteins were not observed (n/o) at the MS analysis stage, but were identified from MS/MS analyses (not available [n/a]). The presence of these proteins in the control experiment, as judged by hypothesis-driven MS/MS analyses, is indicated. When the presence or absence of the protein could not be judged conclusively, due to either depletion of sample or inconclusive fragmentation, the entry is marked as not available (n/a). n/o (not observed) in the score column refers to the proteins (MS) or peptides (MS/MS) that were not assigned a d′ value from the database search using the XProteo software.
(28 KB XLS)

Text S1 Supplementary Materials and Methods, and Supplementary References (78 KB DOC)

## Abbreviations

BAC: bacterial artificial chromosome
CID: collision-induced dissociation
CNS: central nervous system
eGFP: enhanced green fluorescent protein
GFP: green fluorescent protein
MALDI: matrix-assisted laser desorption/ionization
MALDI-IT: matrix-assisted laser desorption/ionization ion trap
MS: mass spectrometry
MS/MS: tandem mass spectrometry
PF/PC synapse: parallel fiber to Purkinje cell synapse
PSD: postsynaptic density
QqTOF: quadrupole/time-of-flight
