# Supplementary figures and images for: Proteomic Studies of a Single CNS Synapse Type: The Parallel Fiber/Purkinje Cell Synapse
Source: PLoS Biol. 2009 Apr 14;7(4):e1000083. doi: 10.1371/journal.pbio.1000083 (PMC2672601; doi:10.1371/journal.pbio.1000083)

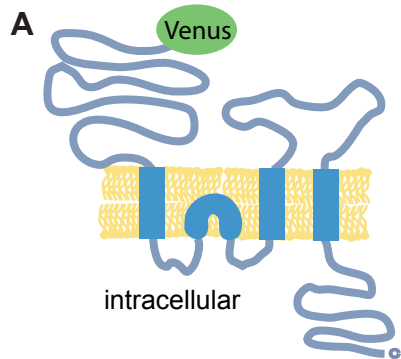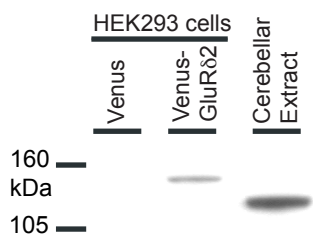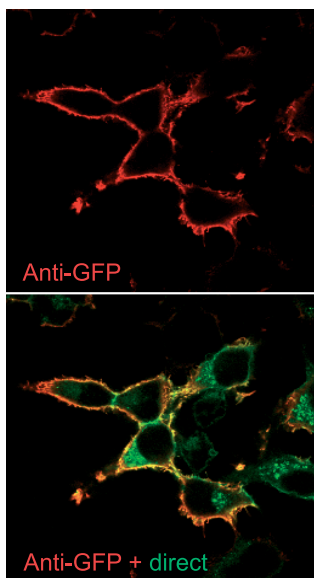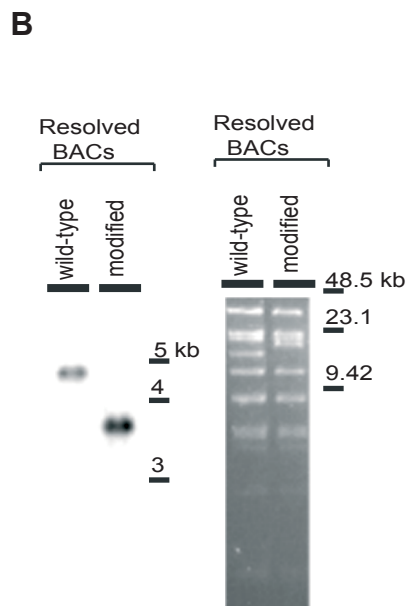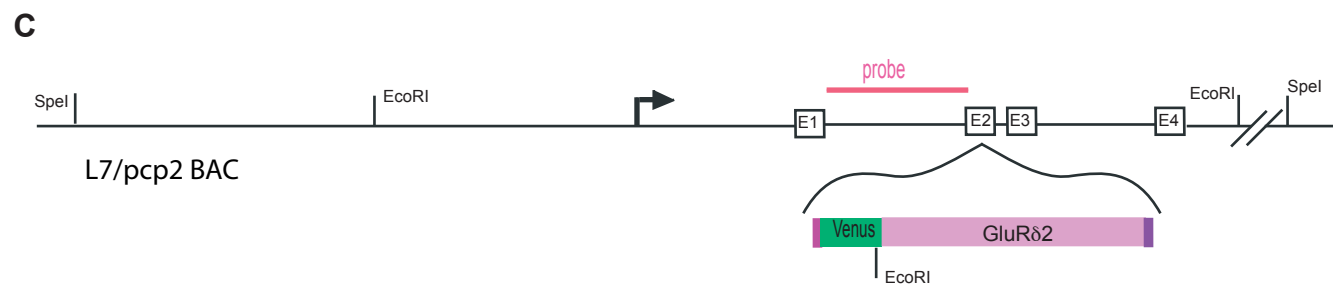

Supplement: Figure S1 — (A) Venus was fused on the N-terminal extracellular part of GluRδ2 (top left panel). A GluRδ2-positive band was detected in protein extracts from VGluRδ2-transfected HEK293 cells, but not in extracts from Venus-transfected cells (bottom left panel). The band was at the expected size (about 140 kDa), higher than the endogenous GluRδ2 detected in cerebellar extracts. Immunofluorescence using an anti-GFP antibody detected the extracellular Venus in VGluRδ2-transfected cells in nonpermeabilizing conditions (red, right panels), showing the proper topography of the tagged receptor. (B) The correct modification of the Pcp2 BAC with the VGluRδ2 construct was checked by Southern blot (left panel, probe shown in [C], BAC DNA digested with EcoRI) and pulse-field gel electrophoresis (right panel, BAC DNA digested with SpeI), before injection in mouse oocytes. (C) Schematic diagram of the BAC containing the Pcp2 gene, known to be expressed specifically in Purkinje cells. The VGluRδ2 cDNA was placed at the level of the Pcp2 ATG. The arrow indicates the promoter region. (463 KB AI). [file pbio.1000083.sg001.pdf]

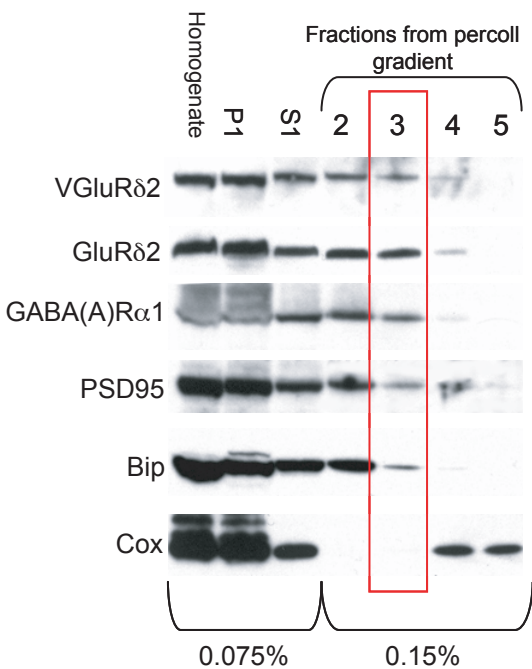

Supplement: Figure S2 — Fractions obtained using the protocol of Dunkley et al. [47] for synaptosome preparation were probed for excitatory synapse markers (GluRδ2, PSD95), the inhibitory synapse marker GABA(A)Rα1, the endoplasmic reticulum marker BiP, and the mitochondrial marker COX. VGluRδ2 was detected using an anti-GFP antibody. (498 KB AI). [file pbio.1000083.sg002.pdf]

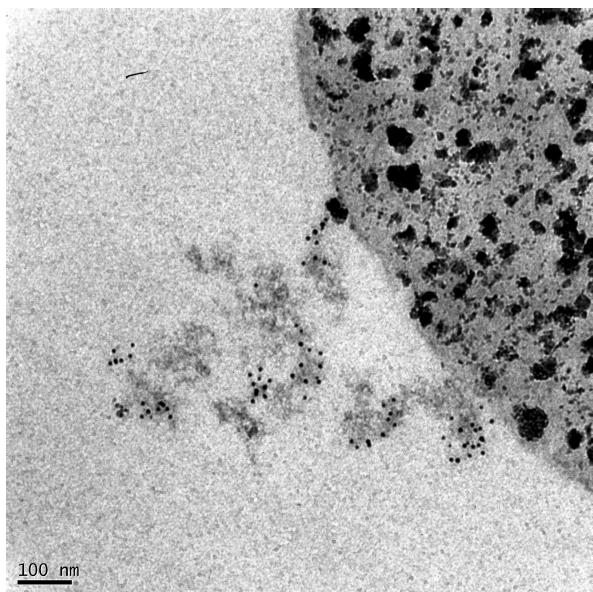

Supplement: Figure S3 — (9.05 MB AI). [file pbio.1000083.sg003.pdf]

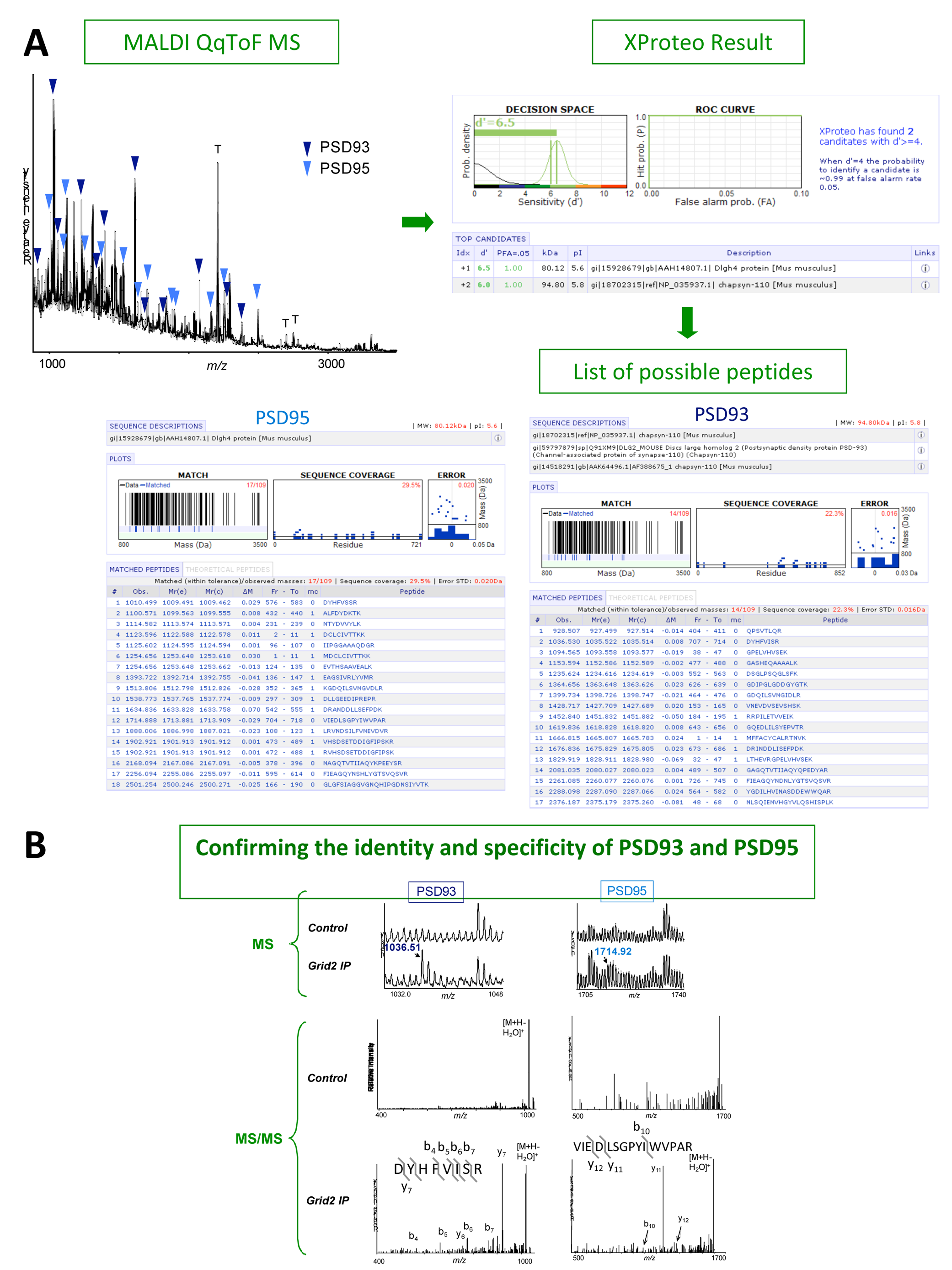

Supplement: Figure S4 — (A) Following in-gel digestion with trypsin, the mixture of peptides was analyzed by MALDI QqToF MS. The m/z values of the [M+H]+ peptides were searched in the NCBI database using the XProteo software, and PSD93 and PSD95 were the first two hits with high scores. The lists of putative peptides generated by the XProteo software are shown, and their presence in the MALDI QqToF MS is indicated. T, trypsin peptides. (B) The identity of the proteins was confirmed using MALDI-IT CID MS/MS analyses, and their specificity of isolation was investigated using a hypothesis-driven tandem MS approach on preparations from Pcp2/eGFP transgenic mice (GFP). Examples of results from MS/MS analyses on peptides of both high- and low-signal-to-noise ratios are shown. (1.55 MB TIF) [file pbio.1000083.sg004.tif]

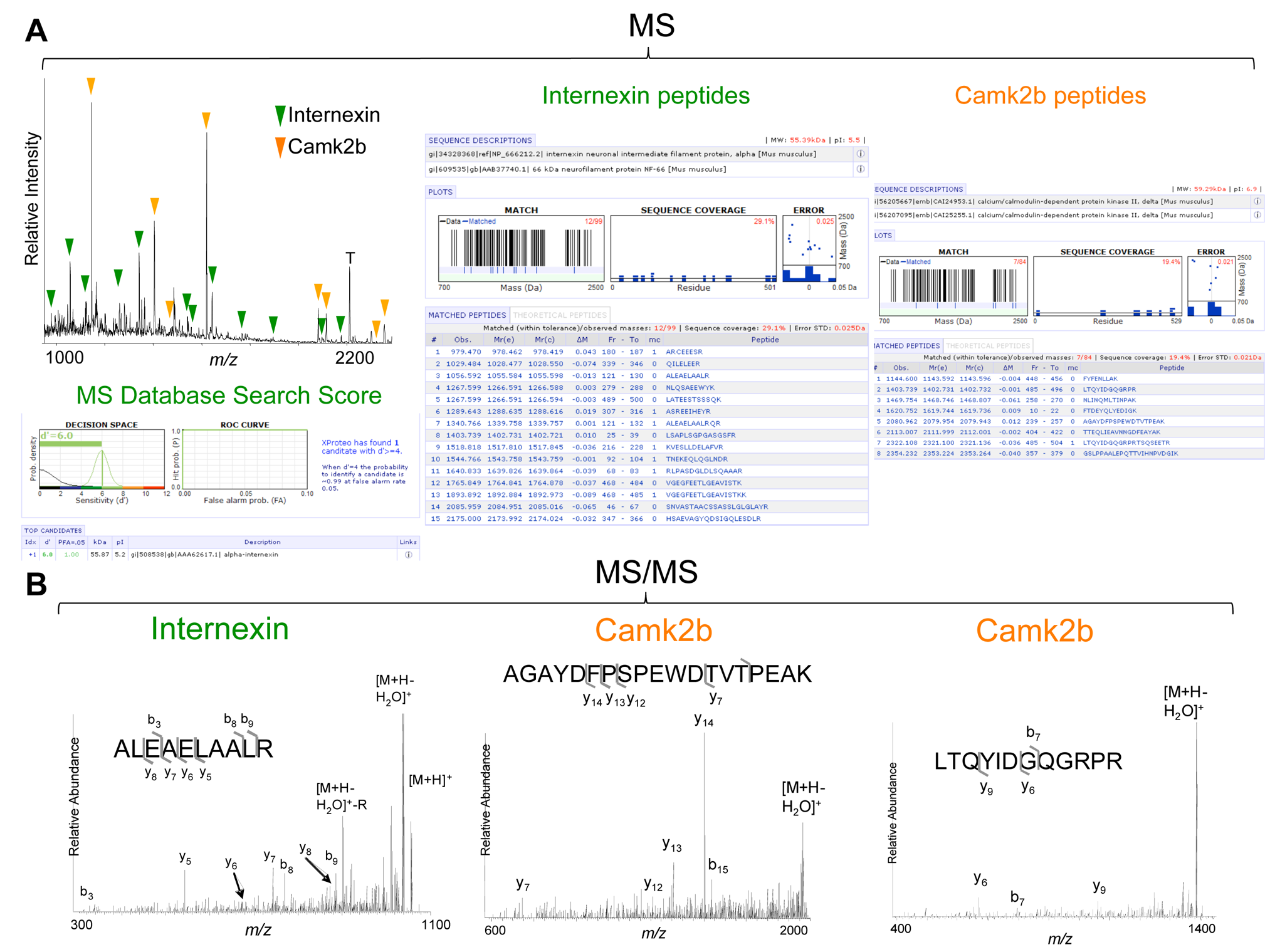

Supplement: Figure S5 — (A) Internexin and Camk2b peptides were both observed following MALDI QqToF MS analysis; however, only internexin received an XProteo database search score (d′ = 6). (B) The presence of both internexin and Camk2b was confirmed using MALDI-IT CID MS/MS analyses. (868 KB TIF) [file pbio.1000083.sg005.tif]

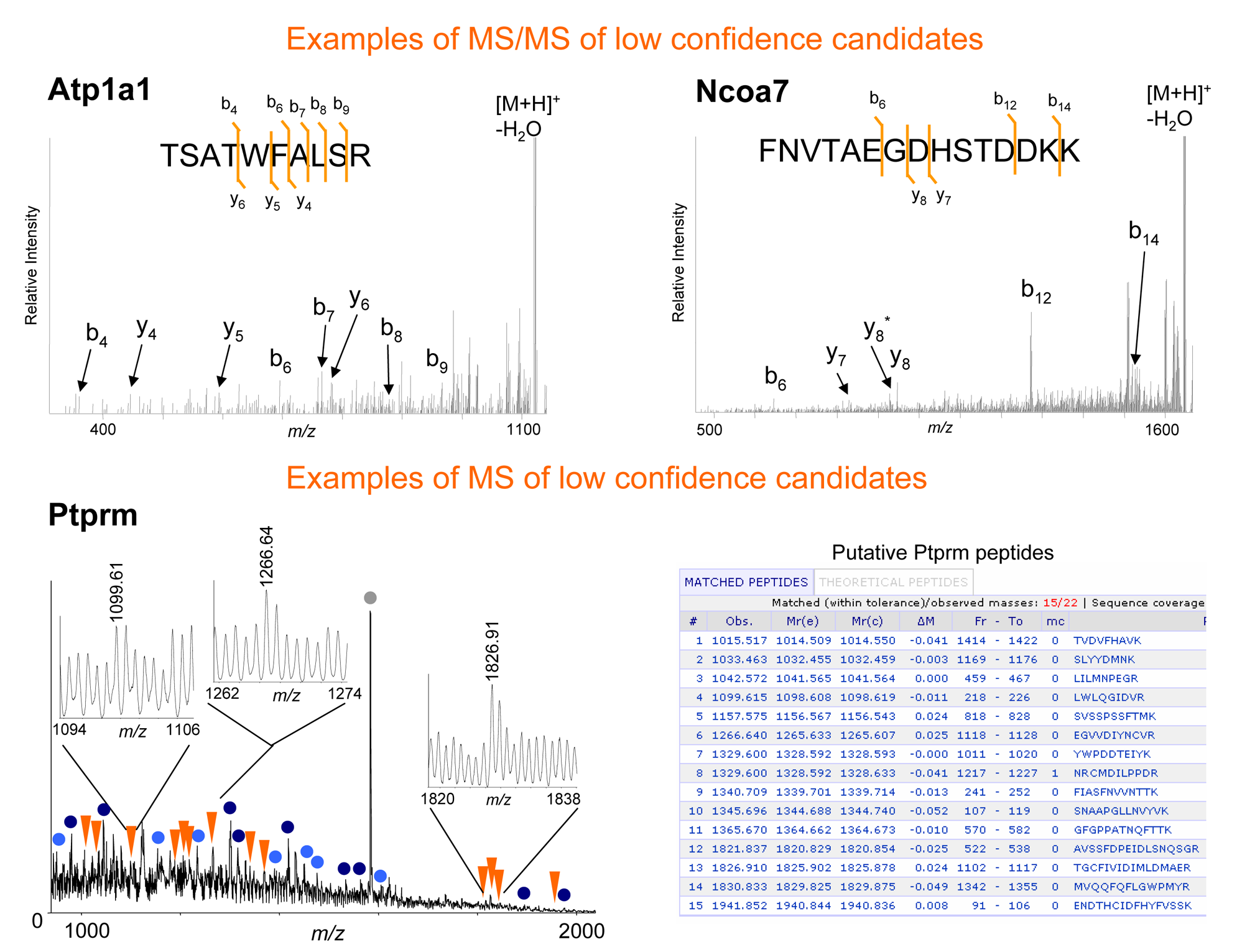

Supplement: Figure S6 — Representative MALDI-IT CID MS/MS spectra are shown for Atp1a1 and Ncoa7. MALDI QqToF MS data and list of putative peptides are illustrated for Ptprm. The peaks attributed to Ptprm are shown with orange arrowheads. This portion of the gel contained multiple proteins. Light blue and dark blue dots indicate selected peaks attributed to Fodrin alpha chain and traces of GluRδ2, respectively. GluRδ2 was primarily identified in another gel band. Grey dot indicates a heavy labeled GluRδ2 peptide, spiked in all samples containing GluRδ2. (823 KB TIF) [file pbio.1000083.sg006.tif]
