# Supplementary material for: Proteomic Studies of a Single CNS Synapse Type: The Parallel Fiber/Purkinje Cell Synapse
Source: PLoS Biol. 2009 Apr 14;7(4):e1000083. doi: 10.1371/journal.pbio.1000083 (PMC2672601; doi:10.1371/journal.pbio.1000083)
Supplement: Text S1 — (78 KB DOC) [file pbio.1000083.sd001.doc]

**SUPPORTING INFORMATION for Selimi et al. 2009**

Supplementary methods [2](#__RefHeading___Toc95561490)

1. Antibodies [2](#__RefHeading___Toc95561491)

2. BAC modification and transgenic mice [3](#__RefHeading___Toc95561492)

3. Protein extracts for expression analysis [4](#__RefHeading___Toc95561493)

4. Western blot [4](#__RefHeading___Toc95561494)

5. Immunofluorescence [5](#__RefHeading___Toc95561495)

6. Database searching using MS and MS/MS mass spectrometric data: [6](#__RefHeading___Toc95561496)

7. MS/MS hypothesis-driven approach: [7](#__RefHeading___Toc95561497)

Supplementary references [8](#__RefHeading___Toc95561498)

Supplementary methods

1. Antibodies

The following antibodies were used (dilution): Rabbit anti-GluRδ2, Chemicon # AB1514 (1/2000); Mouse anti-GluR2, Chemicon #MAB397 (1/500); Mouse anti-PSD95, Affinity Bioreagents #MA1-046 (1/2000); Rabbit anti-PSD93 Chemicon #AB5168 (1/100); Mouse anti-NR2A, Chemicon #MAB5216 (1/500); Mouse anti-Gephyrin, BD transduction Laboratories #610584 (1/250); Mouse anti-GABA(A) receptor β2/3, Upstate #05-474 (1/1000); Rabbit anti- GABA(A) receptor α1, Upstate #06-868 (1/1000); Rabbit anti- GABA(A) receptor α6, Chemicon #AB5610 (1/2500); Rabbit anti-Homer (H-342), Santa Cruz #sc-15321 (1/200); Mouse anti-BiP/GRP78, BD transduction Laboratories #610978 (1/500); Mouse anti-COX (cytochrome oxidase subunit I), Molecular Probes #A6403 (1/20000); Rabbit anti-RPTPmu, Abcam #ab23820; Goat anti-BAIAP2, Abcam #ab15697; Rabbit anti-delta2 Catenin, Abcam # ab11352; Rabbit anti-mGluR1, Abcam #ab6439 (1/1000); Guinea pig anti-VgluT1, Chemicon (1/3000); Guinea pig anti-VgluT2, Chemicon (1/3000); Mouse anti-GAD65/67, Stressgen bioreagents #MSA-225 (1/500); Rabbit anti-synapsin I #AB1543, Chemicon (1/1000); Mouse anti-synaptophysin #611880, BD Biosciences (1/5000).

Rabbit anti-Neph1 was a generous gift from Pr. Sumant Chugh.

The polyclonal anti-Gm941 antibody was custom-generated by injection into rabbits of peptide #1, LKEGDEEIKSDIYTLC, and peptide#2, PLKVERAPAPHGPC. Bleeds were purified using protein G and then affinity-purified against peptide # 2 (Green Mountain Antibodies, Burlington, USA).

The polyclonal anti-MRCK gamma antibody was custom-generated by injection into rabbits of the following peptide: SERPRSLPPDPESESSPC. Bleeds were purified using protein G and then affinity-purified against the peptide (Green Mountain Antibodies, Burlington, USA).

The anti-GFP antiserum was generated by injection in a goat of the full-length GFP (Green Mountain Antibodies) and was affinity-purified using a column made of Sepharose-4B resin coupled to full-length GFP.

1. BAC modification and transgenic mice

The cDNA encoding GluRδ2 together with the 3’UTR was amplified from cerebellar RNA, and placed in frame with a preprotrypsin signal sequence and Venus in a building vector based on eGFP-C2 (Clontech, Mountain View, USA). The sequence encoding the tagged VGluRδ2 and the SV40 polyadenylation signal from the building vector were subcloned into the PL53.SC-AB shuttle vector. The Pcp2 containing BAC RP23-192G13 was then modified by homologous recombination using this shuttle vector and the two-step method (Gong S. et al., 2002, *Genome Res*, **12**:1992). Recombination boxes of 1 kb were amplified from the BAC genomic DNA using the following primers: for box A, 5’TTGGCGCGCCGGTTCCACCCTCATGTTG3’ and 5’AGCTTTGTTTAAACCCGATCGCCCTGCACGTGGGG3’; for box B, 5’ ATAAGAATGCGGCCGCCGGCTTTCTGGGTTCTGGC3’ and 5’ATAAGAATGCGGCCGCGTTTAAGCCAGGTGTGGG3’. These recombination boxes allow the replacement of the Pcp2 ATG by the cDNA construct. Correct modification of the Pcp2 BAC was visualized by southern blot on BAC DNA digested by EcoRI, separated on 0.8% agarose gel and probed with P^32^dATP labeled box A. Pulse field gel electrophoresis was performed on BAC DNA digested by SpeI.

A correctly modified BAC was purified by cesium chloride and DNA was then dialyzed in oocyte injection buffer for generation of transgenic mice. Integration of the BAC in the mouse genome was visualized by southern blot using genomic DNA digested by EcoRI and box A as a probe.

1. Protein extracts for expression analysis

Total protein extracts from cerebellum were prepared by homogenizing the tissue and incubating for 30 minutes at 4°C in a buffer containing 50 mM Tris-Cl, 150mM NaCl, 0.1% SDS, 0.5% sodium deoxycholate and 1% NP-40 complemented with a protease inhibitor cocktail. The homogenate was then sonicated and centrifuged 30 minutes at maximum speed to provide the supernatant for western blot analysis.

For immunoprecipitation experiments, the homogenate from each cerebellum was incubated in 50 mM Tris-Cl, pH=7.4, containing 1% Triton X-100 final for 30 minutes and then centrifuged at maximum speed. The supernatant was affinity-purified using 0.5 mg anti-GFP coated dynabeads for one hour at 4°C. Beads were washed with 50 mM Tris-Cl, pH=7.4, containing 1% Triton X-100 and immunocomplexes eluted for western blot analysis.

1. Western blot

Protein samples (dissolved in NuPAGE LDS sample buffer, Invitrogen, Carlsbad, USA) were separated on 4-12% NuPAGE Bis-Tris gels (Invitrogen). Proteins were then transferred using the semi-dry method (SD transfer cell, Biorad, Hercules, USA) on Immobilon-P PVDF membrane (Millipore, Bedford, USA). Antibodies were diluted in 5% milk/PBS/0.2% tween-20. Secondary antibodies were conjugated to horseradish peroxidase (Pierce) and detection performed using a chemoluminescent substrate.

1. Immunofluorescence

Mice were perfused transcardiacally using 4% paraformaldehyde in phosphate buffer saline pH=7.4 (PBS), then 10% sucrose in PBS. Brains were incubated for 3 days in 30% sucrose in PBS. 25 μm-thick cerebellar sections were cut using a freezing sliding microtome.

For detection of VGluRδ2, sections were incubated in 0.3% H_2_O_2_ in PBS at 4°C, washed in PBS and preincubated in 4% normal donkey serum in PBS. Incubation with the goat anti-GFP antibody (diluted 1/25000 in 1% normal donkey serum/PBS/1%TritonX100/0.1%fish gelatin) was performed overnight at 4°C. Immunolabeling was detected using a biotinylated anti-goat secondary antibody (1/5000 in PBS/1%TritonX100/0.1% fish gelatin) followed by amplification using streptavidin-HRP (1/500) and TSA-FITC (Perkin Elmer, Waltham, USA). All washes were performed in PBS/1%TritonX100.

For detection of the other antigens by immunofluorescence, sections were incubated overnight with the corresponding antibodies and mouse anti-calbindin (1/5000, Swant, Bellinzona, Switzerland) diluted in 1% normal donkey serum/PBS/0.2%TritonX100. Immunolabeling was detected using an Alexa-488 conjugated anti-rabbit or anti-goat and a Rhodamine-RedX conjugated anti-mouse or Cy3 conjugated anti-guinea pig. All washes were performed in PBS/0.2% Triton X-100.

Pictures were taken using a LSM 510 laser scanning confocal microscope (Carl Zeiss, Thornwood, USA).

1. Database searching using MS and MS/MS mass spectrometric data:

We used the XProteo computer algorithm ([www.xproteo.com](http://www.xproteo.com/)) search the NCBI database with MS and MS/MS data. The parameters used for searching the MS data were: Data type: MS; Species: Mus musculus (although for detecting the tag, searches in All entries were performed); NCBI database; protein mass 0-300 kDa; protein pI: 1-14; Mixture Search: Auto; Display top: 20; Enzyme: Trypsin; Max. missed cleavages: 1; Mass type: Monoisotopic; Charge state: MH+; Mass tolerance: 0.03 Da.

Due to the low quantities of sample, the MS/MS CID data was acquired and interpreted manually. This helped limit the number of acquired microscans to that optimal for each peptide fragmentation and it reduced the redundancy in confirming the presence of the more abundant proteins. After manual interpretation, the MS/MS CID data was converted to dta files and also searched in the database using XProteo. The parameters used for searching the MS/MS data were: Data type: MS/MS; Species: Mus musculus; NCBI database; protein mass 0-300 kDa; protein pI: 1-14; Mixture Search: Auto; Display top: 20; Enzyme: Trypsin; Max. missed cleavage: 1; Mass type: Monoisotopic; Charge state: MH+; Precursor tolerance: 0.03Da; Fragment tolerance: 0.6Da; Instrument: MALDI_I_TRAP. Any new assignment made by XProteo was carefully checked manually.

The candidate proteins were scored by XProteo using probability scores calculated with an improved version of the ProFound Bayesian algorithm (Zhang, W. and Chait, B.T., ProFound: an expert system for protein identification using mass spectrometric peptide mapping information. *Anal. Chem.* **72**, 2482-2489 (2000)). The XProteo algorithm then measures the d’ (discriminability) for each candidate protein as the normalized distance between the score distribution (of the candidate protein) and the distribution of randomly matched proteins (in units of standard deviation). A score of d’=4 correspond to a true positive rate of 0.99 and a false positive rate of 0.05.

From our experience, the identities of proteins with d’ > 4 could virtually always be readily confirmed by MS/MS. However, we note that low molecular weight proteins and proteins present as complex mixtures often yielded d’ < 4. We tested many of these putative lower confidence identifications by MS/MS, which often allowed for their subsequent confirmation.

1. MS/MS hypothesis-driven approach:

To confirm the specificity of the isolated proteins, immunoaffinity purifications were also performed on preparations from Pcp2/eGFP transgenic mice (GFP). The sample preparation was identical to that performed for the isolations of VGluRδ2 from Pcp2/VGluRδ2 mice as described in the material and methods section of the manuscript. The analysis of the proteins isolated in the control experiment were performed using MALDI QqToF MS and MALDI IT MS/MS analysis as described for the isolations of VGluRδ2. However, even if a protein was not observed in the control samples using these analyses, we performed additional experiments to probe for their presence at lower levels and the specificity of the observed associations. The approach we utilize is termed hypothesis-driven multistage mass spectrometry and previously described (Kalkum M et al, PNAS 2003 Mar 4;100(5):2795-800). Briefly, we searched in the corresponding control samples for the presence of peptides that we confirmed to correspond to proteins isolated with VGluRδ2. Even if peptides were not observed in the control samples at the MS level, their corresponding *m/z* value ([M+H]^+^) was selected and subjected for CID fragmentation and analyzed using MALDI IT MS/MS analyses. This strategy allows the highly sensitive detection of specific peptides from specific proteins, even if the peptide species cannot be discerned in the primary MALDI-ToF analysis because of insufficient signal-to-noise.

Supplementary references

1. www.brain-map.org

2. www.stjudebgem.org.

3. Yuzaki, M., The delta2 glutamate receptor: 10 years later. Neurosci Res, 2003. 46(1): p. 11-22.

4. Uemura, T., H. Mori, and M. Mishina, Direct interaction of GluRdelta2 with Shank scaffold proteins in cerebellar Purkinje cells. Mol Cell Neurosci, 2004. 26(2): p. 330-41.

5. Shiraishi, Y., et al., Differential expression of Homer family proteins in the developing mouse brain. J Comp Neurol, 2004. 473(4): p. 582-99.

6. Fukaya, M. and M. Watanabe, Improved immunohistochemical detection of postsynaptically located PSD-95/SAP90 protein family by protease section pretreatment: a study in the adult mouse brain. J Comp Neurol, 2000. 426(4): p. 572-86.

7. Roche, K.W., et al., Postsynaptic density-93 interacts with the delta2 glutamate receptor subunit at parallel fiber synapses. J Neurosci, 1999. 19(10): p. 3926-34.

8. Miyagi, Y., et al., Delphilin: a novel PDZ and formin homology domain-containing protein that synaptically colocalizes and interacts with glutamate receptor delta 2 subunit. J Neurosci, 2002. 22(3): p. 803-14.

9. Lambolez, B., et al., AMPA receptor subunits expressed by single Purkinje cells. Neuron, 1992. 9(2): p. 247-58.

10. Lujan, R. and R. Shigemoto, Localization of metabotropic GABA receptor subunits GABAB1 and GABAB2 relative to synaptic sites in the rat developing cerebellum. Eur J Neurosci, 2006. 23(6): p. 1479-90.

11. Moga, M.M. and D. Zhou, Annexin 6 immunoreactivity in select cell populations in the rat brain. J Histochem Cytochem, 2002. 50(9): p. 1277-80.

12. Ichikawa, T., et al., Ca2+/calmodulin-dependent protein kinase II in the rat cerebellum: an immunohistochemical study with monoclonal antibodies specific to either alpha or beta subunit. J Chem Neuroanat, 1992. 5(5): p. 383-90.

13. Ching, G.Y., et al., Overexpression of alpha-internexin causes abnormal neurofilamentous accumulations and motor coordination deficits in transgenic mice. J Neurosci, 1999. 19(8): p. 2974-86.

14. Baba-Aissa, F., et al., Purkinje neurons express the SERCA3 isoform of the organellar type Ca(2+)-transport ATPase. Brain Res Mol Brain Res, 1996. 41(1-2): p. 169-74.

15. Kudo, M., et al., Localization of mRNAs for synaptojanin isoforms in the brain of developing and mature rats. Brain Res Mol Brain Res, 1999. 64(2): p. 179-85.
